# Multiclass Radio Frequency Interference Detection and Suppression for SAR Based on the Single Shot MultiBox Detector

**DOI:** 10.3390/s18114034

**Published:** 2018-11-19

**Authors:** Junfei Yu, Jingwen Li, Bing Sun, Jie Chen, Chunsheng Li

**Affiliations:** School of Electronics and Information Engineering, Beihang University, Beijing 100081, China; yujunfei@buaa.edu.cn (J.Y.); lijingwen@buaa.edu.cn (J.L.); chenjie@buaa.edu.cn (J.C.); lichunsheng@buaa.edu.cn (C.L.)

**Keywords:** radio frequency interference, interference detection and suppression, single shot multibox detector, synthetic aperture radar

## Abstract

Radio frequency interference (RFI) is known to jam synthetic aperture radar (SAR) measurements, severely degrading the SAR imaging quality. The suppression of RFI in SAR echo signals is usually an underdetermined blind source separation problem. In this paper, we propose a novel method for multiclass RFI detection and suppression based on the single shot multibox detector (SSD). First, an echo-interference dataset is established by randomly combining the target signal with various types of RFI in a simulation, and the time–frequency form of the dataset is obtained by utilizing the short-time Fourier transform (STFT). Next, the time–frequency dataset acts as input data to train the SSD and obtain a network that is capable of detecting, identifying and estimating the interference. Finally, all of the interference signals are exactly reconstructed based on the prediction results of the SSD and mitigated by an adaptive filter. The proposed method can effectively increase the signal-to-interference-noise ratio (SINR) of RFI-contaminated SAR echoes and improve the peak sidelobe ratio (PSLR) after pulse compression. The simulated experimental results validate the effectiveness of the proposed method.

## 1. Introduction

With their ability to obtain high-resolution images under all time and weather conditions, synthetic aperture radar (SAR) systems are proven to be very useful methods for earth resource surveys and military reconnaissance. Apart from the target signal, signals received by the SAR system may also have interference from communication devices, television networks, and other radar systems [1,2,3,4,5,6,7,8]. These interference signals are collectively referred to as radio frequency interference (RFI). On the one hand, the energy of RFI is much greater than the energy of the target signal, which may result in the quality of a SAR image being significantly degraded [1,2,3,4,5,6,7,8]. On the other hand, there are diverse forms of RFI due to their different sources [1,2,3]. The multiple types of RFI bury the real target signal and blur the SAR imagery [1,2,3,4,5,6,7,8]. Thus, it is essential to study the characteristics of RFI and develop an interference detection and suppression algorithm for SAR signal processing.

Radio frequency interference detection is the first step towards achieving interference suppression. To achieve this goal, many methods have been proposed to detect RFI in SAR echoes. On the basis of the statistical features of the real target signal, some articles [3,4] have assumed that the spectrum of the radar echoes obeys a complex Gaussian distribution. Based on this, a few statistical test methods have been applied to detect the existence of interference in SAR signals. Some scholars [5,6,7,8,9] proposed measures such as correlation detection or threshold detection in the frequency domain to determine whether the target signal is contaminated by using the characteristics of the contaminated signal in the frequency domain. If accurate prior information is available, the above methods can obtain high accuracy detection results. However, the lack of prior information and inevitable systematic error usually means the accuracy of these methods does not meet the detection requirements.

In recent decades, many RFI suppression algorithms have been studied and developed. Some researchers transformed the signal to a specific domain (e.g., frequency domain and time–frequency domain) and designed a notch filter to remove interference signals with specific bandwidths [8,9,10,11]. Radio frequency interference can be effectively suppressed by these methods. Nevertheless, when the RFI occupies a wide bandwidth, large overlapping between the interference signal and the target signal in the frequency domain results in an intolerable loss of the target signal and discontinuity of the frequency domain data [1,2]. In addition, some researchers characterized the interference signal by establishing a mathematical model and then subtracted it from the echo signal [12,13]. These methods can achieve good performance in terms of interference suppression with accurate estimation of the signal model and parameters, at the cost of an increase in the computational burden and a decline in system efficiency. Other researchers made use of the differences in statistical characteristics between the target signal and the interference signal and put forward some blind source separation algorithms such as feature subspace filtering, independent component analysis (ICA), independent subspace analysis (ISA), and adaptive filtering [3,4,5]. When the target signal is not correlated with the interference signal, these methods can effectively mitigate the interference. However, in actual situations, the compositions of the target and interference signals are extremely complex, and so their statistical characteristics have difficulty satisfying the unrelated conditions, which results in these methods having limited effectiveness.

Many scientific experiments have demonstrated that deep learning networks have an impressive ability to acquire data information and detect targets accurately [14,15,16,17,18,19,20]. Compared with traditional technology, the advantage of deep learning lies in its capability to extract deep features of data adaptively and achieve classification or target detection through multilayer neural networks without any prior knowledge [18,19,20]. This merit has led to the widespread use of deep learning in remote sensing and SAR image processing [21,22,23,24]. However, as far as we know, the single shot multibox detector (SSD) [20], an efficient and practical target detection algorithm, has not been applied to the detection, classification, or parameter estimation of RFI. We believe that the following reasons may have led to this. First, compared with the one-dimensional SAR echo data, the SSD is more sensitive to two-dimensional image data, which makes it arduous for SSD to identify the interference from the echo data without any preprocessing. Second, when the SAR images are covered by interference, it is difficult for SSD to extract useful information about the target and interference from them. Finally, to the best of our knowledge, there is no public dataset containing RFI and echo signals to train an SSD.

To solve these problems, we propose a new intelligent detection, identification, and suppression method for multiclass RFI signals based on the SSD. First, this method establishes the signal model of the echo pulse and generates a dataset containing interference-contaminated echo signals through simulation. Additionally, the time–frequency form of the dataset is obtained by the short time Fourier transform (STFT). Then, the proposed method employs the time–frequency domain dataset as input data to the SSD for network training. An SSD network capable of simultaneously detecting, identifying, and estimating the interference signal in the SAR echo is obtained. Through this network, we not only predict the existence of interference in the unknown echo signal, but also identify its type and estimate its related parameters. Then, the predicted interference signal can be established according to the prediction result. Finally, an adaptive filter is used to get the optimal estimation of RFI and remove it from an echo signal.

The rest of this article is organized as follows. Section 2 formulates the signal model of the target signal, the interference, and the echo signal. Section 3 discusses the proposed RFI detection and suppression method. The simulation experiment is detailed in Section 4. Section 5 summarizes the article.

## 2. Signal Model

For a normal SAR system, the signal received by each echo window includes the target, RFI and noise signals [1,2,3,4,5,6,7,8]. Therefore, the contaminated echo signal can be modeled as:

(1) S(τ)=R(τ)+I(τ)+N(τ),

where τ denotes the fast time, and R(τ), I(τ), and N(τ), respectively, denote the target, RFI, and noise signals. It is important to note that the noise of the system is approximated by Gaussian noise.

Since the SAR system usually works in a complex electromagnetic environment, the form of the interference in the echo is complicated and difficult to model. In general, RFI is classified into narrowband interference (NBI) and wideband interference (WBI) based on the bandwidth of the interference signals. The bandwidth of NBI is generally less than 1% of the transmitted signal [1,2,3,4,5], while the bandwidth of WBI is larger than 10% of the transmitted signal [1,2]. Based on this, researchers often establish a model of the jamming signal using either a linearly-modulated [1,25] or a sinusoidally-modulated [1,2,3,4,5] model. This modeling method has achieved good results in interference suppression, but it ignores the situation where both forms simultaneously exist. Considering that broadband interference is generally derived from other radar systems, it can be modeled with a linear frequency modulated signal. However, the source of NBI is particularly complex. If it is modeled as a single form, it cannot include the multiple interferences that SAR is subjected to. Therefore, this paper proposes to model NBI with a sum of sinusoidally-modulated radio frequency noise interference and a linear frequency modulated NBI. Such a modeling method will make the established interference signal model more accurate and improve the effect of interference suppression.

In summary, the interference signal in this paper is modeled as the sum of the radio frequency noise interference signal (RFNI), the narrowband linear frequency modulated interference signal (NBLFMI), and the wideband linear frequency modulated interference signal (WBLFMI), which can be written as:

(2) $$\left\{\begin{array}{cc} I(\tau ) & =I_{RFNI}\left(\tau \right)+I_{NBLFMI}\left(\tau \right)+I_{WBLFMI}\left(\tau \right),\\ I_{RFNI}\left(\tau \right) & =\sum_{i=1}^{L_{I}}A_{i}\left(\tau \right)rect\left(\tau /T_{i}\right)exp\left\{j\left[2\pi f_{i}\tau +\varphi_{i}\right]\right\},\\ I_{NBLFMI}\left(\tau \right) & =\sum_{n=1}^{L_{N}}A_{n}\left(\tau \right)rect\left(\tau /T_{n}\right)exp\left\{j\left[\pi k_{n}\tau^{2}+\varphi_{n}\right]\right\},\\ I_{WBLFMI}\left(\tau \right) & =\sum_{m=1}^{L_{M}}A_{m}\left(\tau \right)rect\left(\tau /T_{m}\right)exp\left\{j\left[\pi k_{m}\tau^{2}+\varphi_{m}\right]\right\}, \end{array}\right.$$

where $I_{RFNI}\left(\tau \right)$, $I_{NBLFMI}\left(\tau \right)$, and $I_{WBLFMI}\left(\tau \right)$, respectively, denote the RFNI, NBLFMI, and WBLFMI signals; $A_{i}\left(\tau \right)$, $A_{n}\left(\tau \right)$, and $A_{m}\left(\tau \right)$ denote the envelopes of the interference signals; $T_{i}$, $T_{n}$, and $T_{m}$ denote the pulse widths; $\varphi_{i}$, $\varphi_{n}$, and $\varphi_{m}$ represent the phase; $f_{i}$ denotes the frequency of the RFNI; $k_{n}$ and $k_{m}$ are the chirp-rates of the NBLFMI and WBLFMI; and $L_{I}$, $L_{N}$, and $L_{M}$ represent the amounts of three types of interference signals.

## 3. Interference Detection and Suppression Method

**Figure 1:**
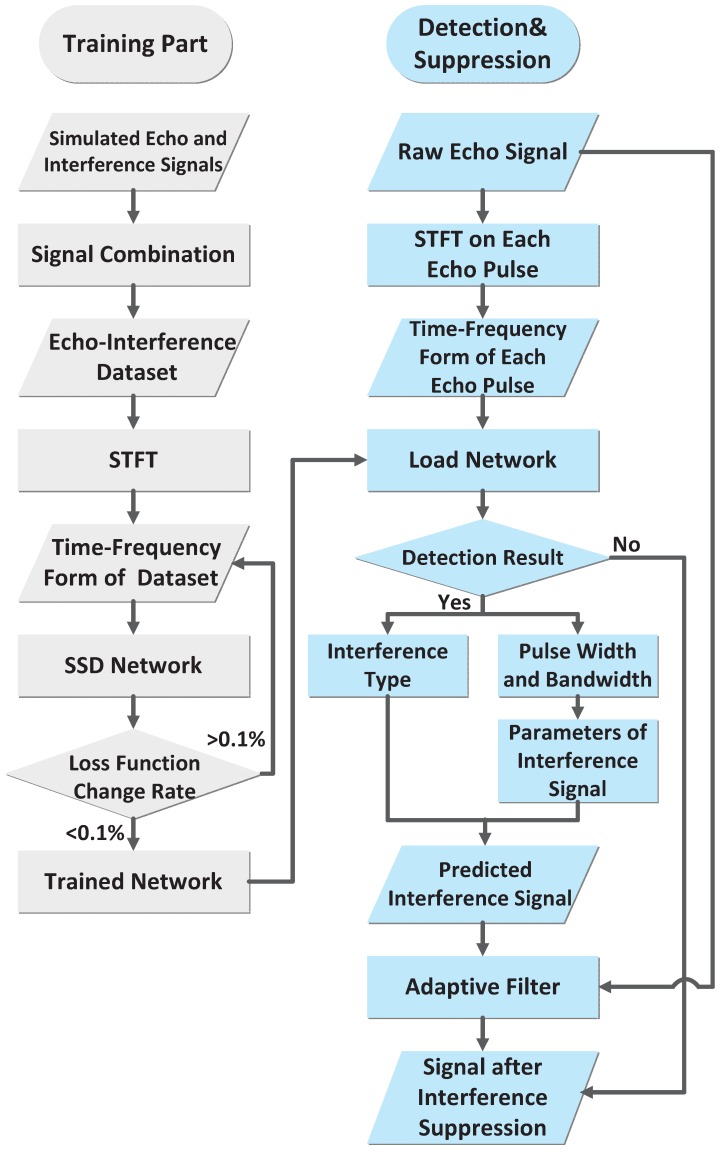
Flowchart of the proposed interference detection and suppression method. STFT indicates the short-time Fourier transform, SSD indicates the single shot multiBox detector.

This section introduces the interference detection and suppression method proposed in this paper. First, according to the above signal model and statistical characteristics, an echo signal dataset containing interference signals is obtained through a simulation. Additionally, the short-time Fourier transform is used to get its time–frequency domain form. A deep learning network with the ability to perform interference signal detection, identification, and parameter estimation is trained with the time–frequency dataset as the input. After the training has been completed, an unknown signal is put into the network to check if interference exists. If it exists, the network will give the type of the interference and calculate its parameter estimation results. Based on this, the predicted interference signal is obtained. Finally, an adaptive filter is used to acquire the signal after interference suppression. The process is summarized in Figure 1.

### 3.1. Dataset Preparation

#### 3.1.1. Simulation of the Time-Domain Dataset

To accurately obtain information about the interference signal from the echo, many data are required to train the deep learning network. However, there is currently no published dataset of measured SAR echo signals containing RFI. Based on the model and statistical characteristics of the signals described in the second section, we propose generating data through simulations. A dataset that satisfies the features of the real target signal can be obtained by setting a series of reasonable parameters.

For the target signal, the scattering point model shows that the statistical properties of the complex-valued SAR image for a uniform scattering scene can be expressed as [26,27,28]:

(3) Z=Aexp{jθ},

where *Z* denotes the complex-valued SAR image, *A* denotes the amplitude of the image, and θ is the phase of the image. The amplitude and phase are independent. Considering the antenna pattern and the changes of different scenes, many statistical experiments have shown that the amplitude of the SAR image obeys certain statistical models [26,27,28]. Based on the ideal assumption that the imaged scene has a constant radar cross section (RCS), *A* obeys the Rayleigh distribution and θ obeys a uniform distribution. Based on a more realistic assumption, *A* obeys the K distribution and θ obeys a uniform distribution. In addition, to ensure that the simulated echo signal has the characteristics of the radar transmit signal, the demodulated echo signal can be modeled as a convolution of the complex-valued SAR image with the radar transmit signal (e.g., chirp signal):

(4) $$X\left(\tau \right)=Aexp\left\{j\theta \right\}⊗rect\left(\tau /T\right)exp\left\{j\pi k\tau^{2}\right\},$$

where rect(·) is the rectangular window function, *T* is the pulse width of the chirp signal, and *k* is the chirp rate of the chirp signal. In addition, other parameters of the echo signal, such as the sampling frequency, the width of the echo window, and signal–to–noise ratio, need to be determined.

Equation (2) gives the signal model of the RFI. To make the simulated interference signal as similar as possible to the signal in the actual situation, we must create a sufficient number of interference signals with various parameters. Therefore, this paper proposes to set the parameters—such as the amplitude, frequency, chirp rate, and phase of the signal in Equation (2)—to random variables that obey certain distributions in order to generate multiple sets of interference signals. On this basis, the model of interference signal in Equation (2) is further written as:

(5) $$\left\{\begin{array}{cc} I_{RFNI}\left(\tau \right) & =A_{R}\left(\tau \right)rect\left(\left(\tau -\tau_{R}\right)/T_{R}\right)exp\left\{\left[2\pi f_{R}\left(\tau -\tau_{R}\right)+\varphi_{R}\right]\right\},\\ I_{NBLFMI}\left(\tau \right) & =A_{N}\left(\tau \right)rect\left(\left(\tau -\tau_{N}\right)/T_{N}\right)exp\left\{j\left[\pi k_{N}{\left(\tau -\tau_{N}\right)}^{2}+\varphi_{N}\right]\right\},\\ I_{WBLFMI}\left(\tau \right) & =A_{W}\left(\tau \right)rect\left(\left(\tau -\tau_{W}\right)/T_{W}\right)exp\left\{j\left[\pi k_{W}{\left(\tau -\tau_{W}\right)}^{2}+\varphi_{W}\right]\right\}, \end{array}\right.$$

where $A_{i}\left(\tau \right)$, $\varphi_{i}$, $\tau_{i}$, and $T_{i}$, respectively, represent the envelope, phase, time shift and pulse width of the respective signal; $f_{R}$ denotes the frequency of RFNI; and $k_{i}$ denotes the chirp rate of the respective (narrow or wide band) signals. In this expression, all parameters obey a uniform distribution, except for $A_{R}\left(\tau \right)$, which obeys the Rayleigh distribution.

Based on the above discussion, we can simulate multiple sets of target signal and interference signal data by setting reasonable parameters. For each target signal pulse, multiple interference signals are randomly selected and superimposed to obtain echo pulses containing interference signals, and a time-domain dataset is eventually obtained. It is noteworthy that this paper does not study interferences that act through azimuth lines.

#### 3.1.2. Short-time Fourier Transform

Short-time Fourier transform is an important tool for time–frequency analysis, which can analyze the time–frequency characteristics of time-varying non-stationary signals. Its calculation process can be written as:

(6) $$D_{STFT}\left(m,n\right)=\sum_{k=0}^{N-1}d\left(k\right)\cdot g^{*}\left(k-m\right)exp\left(-2\pi nk/N\right),$$

where $D_{STFT}\left(m,n\right)$ is the time–frequency spectrum of the signal; $g^{*}\left(\cdot \right)$ denotes the conjugate form of the window function; *m* and *n*, respectively, denote the sampling points in the time domain and in the frequency domain; and *N* denotes the total number of samples. As an effective tool for analyzing the time-varying and non-stationary signals, STFT provides time-located spectrum information. Since both the target signal and the interference signal are time-varying and non-stationary, to obtain a comprehensive time–frequency spectrum, STFT is utilized to transform the existing time-domain data into the time–frequency domain and obtain time–frequency graphs (TFG) of all signals. In this way, the time–frequency domain dataset is obtained. It is worth noting that setting different window lengths will cause changes in the temporal resolution and frequency resolution of the TFG, which can affect the possibility of signal detection in the TFG. Therefore, it is necessary to select an appropriate temporal resolution and frequency resolution to ensure the quality of the TFG is sufficient.

#### 3.1.3. Feature Boxes

**Figure 2:**
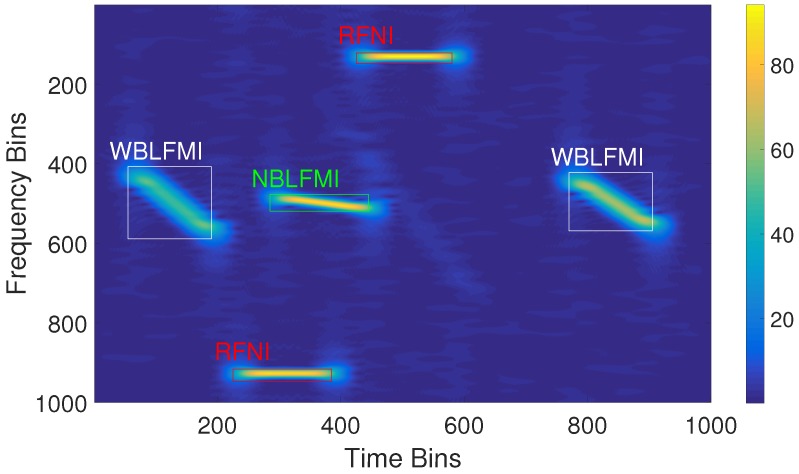
Feature boxes in the time–frequency graph. RFNI indicates the radio frequency noise interference signal, NBLFMI indicates the narrowband linear frequency modulated interference signal, and WBLFMI indicates the wideband linear frequency modulated interference signal.

In the TFG of the signal defined in this paper, the horizontal axis represents time and the vertical axis represents frequency. Thus, feature boxes, which show the time–frequency characteristics and the type of interference signal, can be labeled in the time–frequency domain. Figure 2 shows the results using the set parameters for all of the dataset signals in the time–frequency domain. After labeling, we refer to the time–frequency domain as the echo-interference dataset. The deep learning network proposed in the latter part of this paper takes this echo-interference dataset as its input and extracts the deep feature information of all signals to predict, identify, and estimate the interference.

### 3.2. Detection, Recognition, and Parameter Estimation for the Interference

**Figure 3:**
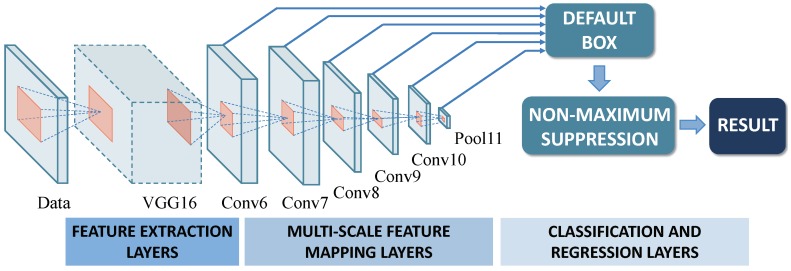
The SSD network structure used in this article.

To accurately detect, identify, and estimate the interference, we first need to extract valid information from the TFG of the echo signal. A target detection network using the SSD was chosen to achieve this goal. The SSD is a real-time multiclass target detector based on a convolutional neural network. To begin with, the network takes images and the feature boxes of the target objects in the images as input, and then extracts the features of the images and the target objects through the VGG16 network(a convolutional neural network). Further, the network sets feature windows of various scales (referred to as default boxes) to match the shapes of the target objects and calculates the positional offset and confidence of each default box by multiscale feature mapping layers. Finally, the detection results related to the targets’ positions are obtained by using a non-maximal suppression algorithm. The framework of the SSD used in this article is shown in Figure 3.

The steps of the SSD network for detecting the signal type and estimating the parameters of the interference in this paper are as follows:

#### 3.2.1. Training Process

1. Extracting the primary feature maps from the data through feature extraction layers:
  The convolutional neural network VGG16 is used as the feature extraction layer in this paper. It can map raw data to high-dimensional space. According to the time and frequency characteristics of the SAR echo signal, this paper makes slight adjustments to the traditional VGG16 network (for example, the kernel size and the kernel initialization) to enhance its ability to extract the features of the SAR echo signal. The details of the network structure are shown in Table 1. After multiple convolution and maxpool operations with this network, primary feature maps that reflect the time–frequency characteristics of the echo are obtained. The subsequent classification and regression steps are performed on these primary feature maps.
2. Extracting advanced feature maps from the primary feature maps through multiscale feature mapping layers:
  The primary feature maps obtained in the previous step are passed through the multiscale feature mapping layers, where each layer uses convolution or pooling to obtain advanced feature maps on multiple scales.
3. Determining feature information through the classification and regression layers:
  The predicted category, the confidence values, and the offsets relative to the system’s default bounding boxes are given for each position of the advanced feature maps. Then, all of these intermediate results are merged using non-maximum suppression. In the end, the final result is delivered to the loss function. As the number of training iterations increases, the value of the loss function decreases. When it reaches a certain value and its change rate is small enough, the network training is considered to be complete. At this time, the network is capable of interference type classification and feature box prediction.

#### 3.2.2. Testing Process

1. Using the network for predictions:
  The time–frequency graph of an unknown signal is obtained by STFT, and it acts as the input to the above SSD network. The network gives the prediction result. The prediction result contains: (1) whether the signal is contaminated; and (2) if contaminated, the interference type and the position of the interference in the time–frequency graph.
2. Estimating interference signal parameters by using the prediction result:
  We can get the predicted feature boxes of the unknown signal with the trained SSD network, and then the signal parameters of the interference can be estimated. The time shifts and pulse widths can be obtained by using the time span of the feature box on the time axis. We can get the frequency and bandwidth of the interference signals by conducting the same operation on the frequency axis. Finally, the predicted interference signal is obtained by Equation (5).

### 3.3. Interference Suppression Method Based on the Adaptive Filter

**Figure 4:**
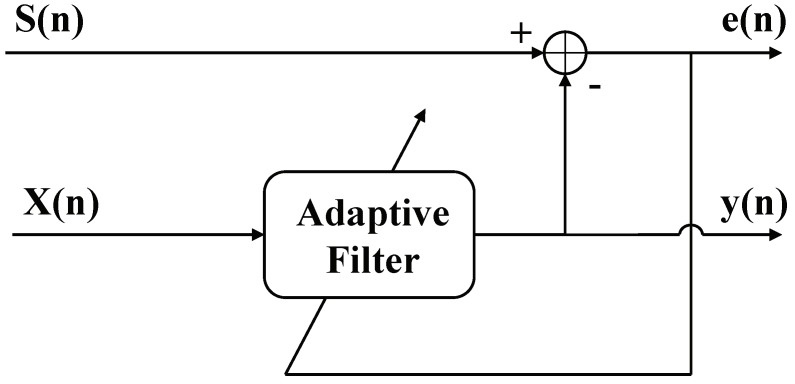
Schematic diagram of the adaptive filter.

The function of the adaptive filter is to continuously adjust the weight coefficients of the filter so that the error of the expected signal and the output signal of the filter satisfy a certain optimal condition, which makes the output signal close to the expected signal [29]. The schematic diagram of the adaptive filter used in this paper is shown in Figure 4.

In this paper, we assume that the weight coefficient vector of the adaptive filter is W(n). The interference signal predicted in the previous step is used as the filter input signal vector X(n). The original echo signal S(n) acts as the expected signal of the filter. The output signal is defined as y(n), and the error signal is defined as e(n), which can be written as:

(7) $$\left\{\begin{array}{ll} y(n) & =W^{T}\left(n\right)X\left(n\right),\\ e(n) & =S\left(n\right)-W^{T}\left(n\right)X\left(n\right). \end{array}\right.$$

When the filter converges to its Wiener filter coefficients, the quadratic of the error between the output of the filter and the desired signal reaches a minimum. Hence, the output signal is the optimal estimation of the interference signal under the condition of minimum mean square error. The error signal is the echo signal after interference suppression. At this time, the weight coefficient vector of the filter is $\hat{W}\left(n\right)$. The interference signal predicted by filter is $\hat{y}\left(n\right)$. The echo signal after interference suppression is $\hat{R}\left(n\right)$. All of which can be written as:

(8) $$\left\{\begin{array}{ll} \hat{w}\left(n\right) & =R_{xx}^{-1}\cdot R_{xd},\\ \hat{y}\left(n\right) & ={\hat{W}}^{T}\left(n\right)\cdot X\left(n\right),\\ \hat{R}\left(n\right) & =S\left(n\right)-\hat{y}\left(n\right), \end{array}\right.$$

where $R_{xx}$ denotes the autocorrelation matrix of the input signal, and $R_{xd}$ is the cross correlation matrix of the input signal and the desired signal. Hence, the steps required to detect and suppress the interference signals for a single pulse are complete. The echo signal after interference suppression can be obtained by repeating the process described above in a pulse-by-pulse manner.

## 4. Simulation Experiments

### 4.1. Dataset Generation and Network Training

In accordance with Equations (4) and (5), the system parameters for the simulation were set as shown in the following tables. Among them, Table 2 shows the simulation parameters for the echo signals and the SAR system. Table 3 gives the parameters for the simulation of the three types of interference signals. It should be noted that the Rayleigh distribution and K distribution were applied to generate the echo signals. Finally, the generated echo signals obey these two distributions with an amplitude in the range [0, 20] dB.

**Figure 5:**
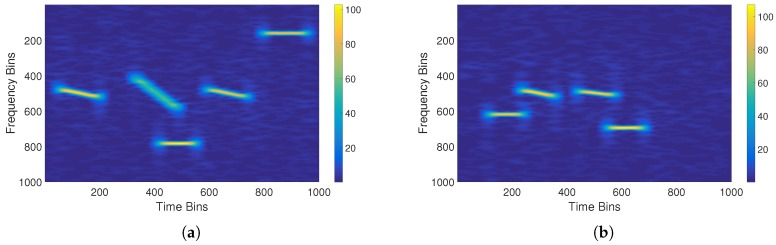
Time–frequency graphs based on two distributions: (**a**) the Rayleigh distribution; and (**b**) the K distribution.

According to the above parameters, 1000 groups of target signals, RFNI signals, NBLFMI signals, and WBLFMI signals were obtained by simulation. To obtain contaminated echo signals, a plurality of sets of interference signals was randomly selected and superimposed on each target signals. Through this operation, an echo-interference time domain dataset was formed by combining all of the simulated echo pulses. After STFT, a time–frequency domain dataset was generated. The temporal resolution and the frequency resolution of the STFT were 0.505 μs and 1.980 MHz. The feature boxes indicating the interference signals were labeled according to the simulation parameters. Figure 5 shows a TFG generated by the Rayleigh distribution and Figure 5 shows one generated by the K distribution.

**Figure 6:**
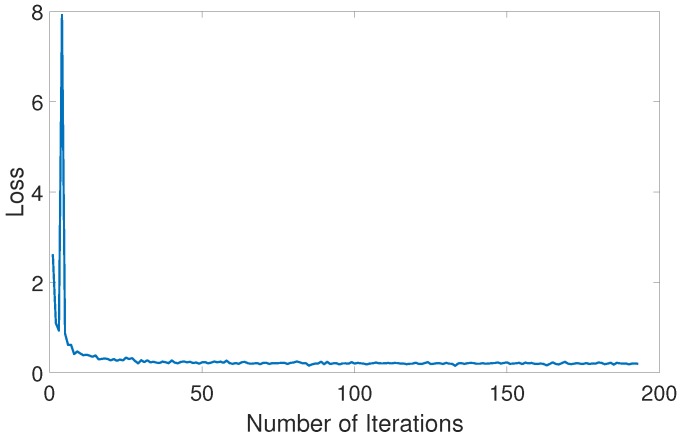
Change of loss function during training.

Before network training began, it was necessary for the time–frequency domain dataset to be randomly divided into training and test sets in a ratio of 8:2. Then, the training set and its feature boxes were put into the SSD network for training, and the value of the loss function was obtained. Figure 6 shows the change of loss function during training. It can be seen that the value of the loss function decreases gradually as the number of training iterations increases. When its absolute value and change rate are very small, this indicates that the training has been completed. The trained network can be used to detect, identify, and estimate the interference.

### 4.2. Simulation Results

#### 4.2.1. Results of the Single Pulse Echo Signal

**Figure 7:**
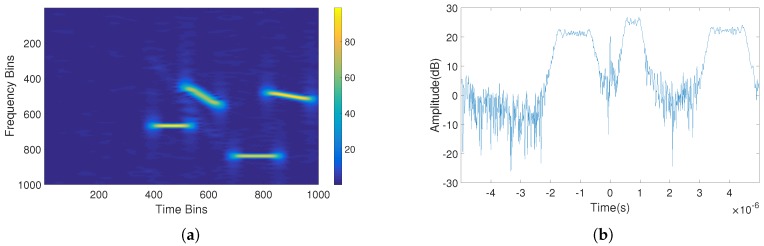
The time–frequency graph and the result of pulse compression for an unknown echo signal: (**a**) the time–frequency graph; and (**b**) the pulse compression result.

To illustrate the effectiveness of the proposed method, this part simulates the interference suppression process of a contaminated SAR echo pulse, and quantitative evaluation metrics are presented. In practical cases, the electromagnetic situation is very complicated, and there may be multiple types of interferences simultaneously in one radar pulse. Therefore, we simulated the situation where three types of interference exist in one echo pulse at the same time, and used the proposed method to perform detection, identification, and parameter estimation of the interference signal. Finally, we obtained the signal after interference suppression. The simulated echo signal was contaminated by two RFNI, one NBLFMI and one WBLFMI signals. The signal-to-interference-noise ratio was approximately −25.02 dB. Figure 7a,b shows the time–frequency graph and the result of pulse compression for the echo signal, respectively. As can be seen in these figures, the target signal was completely submerged in the interference signal with strong power, and even after the pulse compression process, it is still difficult to detect the peak of the target signal from the compression result.

**Figure 8:**
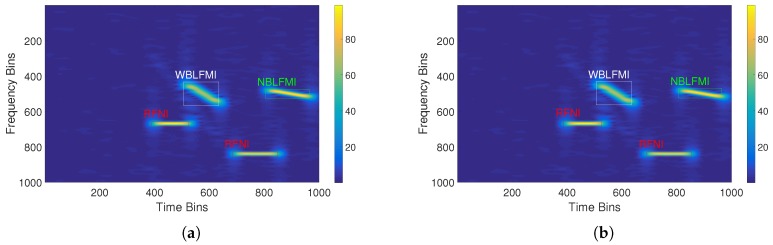
(**a**) The true feature boxes; and (**b**) the network’s prediction boxes for the simulated test echo signal.

As the real echo signal used in this simulation experiment was a single pulse signal, we added a transmitted signal (chirp signal) and an interference signal to obtain the echo signals, and used the STFT to obtain TFGs of them. Then, the SSD network was trained by these TFGs. The time–frequency graph of the signal in Figure 7 was input to the trained network, and the prediction result of the interference was determined. Figure 8 shows the true feature boxes of the signal and the network’s prediction boxes.

The prediction results show that there are two RFNI, one NBLFMI, and one WBLFMI in the echo signal. The simulation not only successfully predicted the existence and type of the interference signals, but also their positions on the time–frequency graph. From this, the predicted parameters for the interference signals were acquired from the coordinates of the feature boxes on the time axis and the frequency axis. The estimated parameters were calculated and are shown in Table 4.

**Figure 9:**
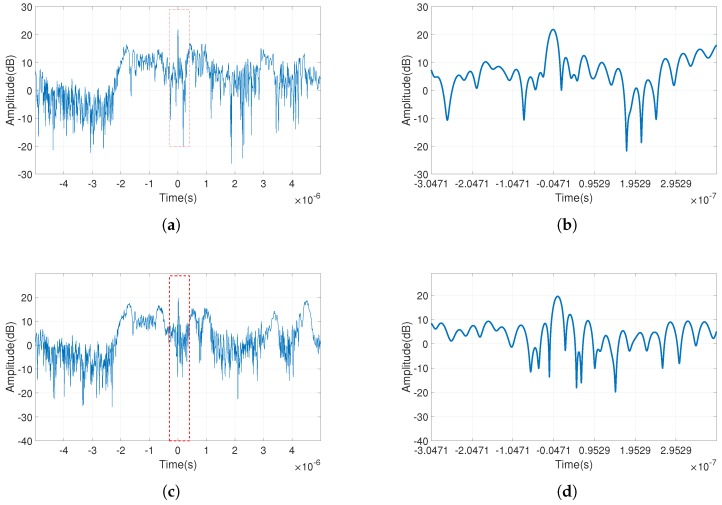
Comparison of pulse compression results obtained via different methods: (**a**) proposed method; (**b**) zoom in on the red box in (**a**); (**c**) time–frequency filtering method; and (**d**) zoom in on the red box in (**c**).

The predicted interference signal was calculated with these estimated parameters, in accordance with Equation (5), and interference suppression was performed with an adaptive filter. Figure 9 compares the interference suppression performance of the proposed method with that of the time–frequency filtering method. The pulse compression results of the signal after interference suppression by the two methods are shown in Figure 9a,c. Figure 9b,d shows the enlarged images of the marked portions of Figure 9a,c, respectively. It can be seen that the time–frequency filtering algorithm has a lower main lobe and a higher sidelobe than the proposed method which means that it loses some information of the target signal.

Two evaluation metrics, the signal-to-interference-noise ratio (SINR) and the peak–to–sidelobe ratio (PSLR), were used to evaluate the effect of interference suppression through a quantitative analysis. Signal-to-interference-noise ratio reflects the ratio of the energy of target signal to the energy of interference and noise. It is defined as:

(9) $$SINR=10log_{10}\left(\frac{{\sum |R\left(\tau \right)|}^{2}}{\sum |S\left(\tau \right)-\hat{R}{\left(\tau \right)|}^{2}}\right),$$

where R(τ) represents the target signal, $\hat{R}\left(\tau \right)$ denotes the estimated target signal, and S(τ) is the contaminated signal. Peak–to–sidelobe ratio measures the ratio of the peak of the highest secondary lobe to the peak of main lobe, and it can be written as:

(10) $$PSLR=20log_{10}\left(\frac{V_{s}}{V_{m}}\right),$$

where $V_{s}$ and $V_{m}$ denote the amplitude of the highest secondary lobe peak and the main lobe peak, respectively.

Table 5 compares these evaluation metrics in different situations. Since the energy of the interference was much larger in the simulation than in the target signal, it was difficult to distinguish the main lobe and the sidelobe of the compressed signal even with the pulse compression process, and it was impossible to calculate the PSLR at this time. In the comparison of the two interference suppression algorithms, it can be seen that, after using the proposed method, the SINR of the final signal improved by 0.826 dB. Additionally, after pulse compression, its PSLR improved by 2.607 dB, and was closer to the corresponding value of the interference-free signal −10.813 dB. The proposed method achieved a satisfactory result in virtue of accurate parameter estimation and effective interference suppression. Comparatively speaking, even knowing exact location of the interference on the TFG, the time–frequency filtering method produced a worse effect due to a large loss of useful signals. In summary, compared with the time–frequency filtering method, the proposed method not only can effectively suppress the interference, but also has a better pulse compression result.

#### 4.2.2. Results of the Distributed Target Echo Signal

**Figure 10:**
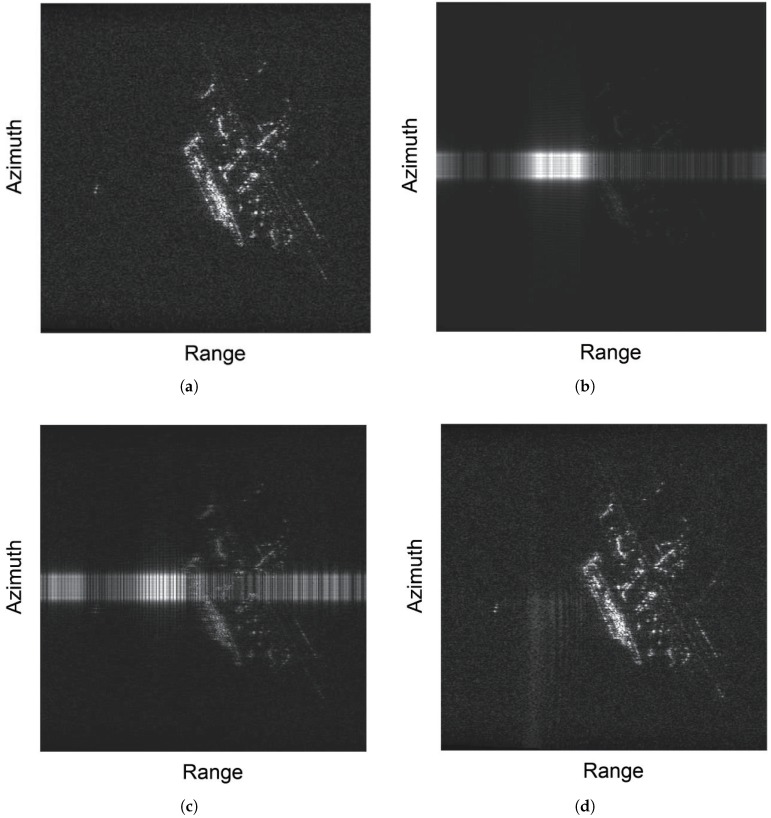
Comparison of images obtained via different methods: (**a**) image without interference; (**b**) image with interference; (**c**) image with time–frequency filtering method; and (**d**) image with the proposed method.

This section describes the use of a simulated distributed echo signal to illustrate the validity of the proposed method. In the simulation, we took the pixel value of a real SAR image as the backscattering coefficient of the scene, and then set reasonable radar and antenna parameters, finally calculating the corresponding echo signals pulse by pulse. The area in the picture was a partial area of a harbor. The bandwidth and the wavelength of the emitted signal were 50 MHz and 0.03 m, respectively. The flight height and velocity of the SAR are 5 km and 200 m/s, respectively. Figure 10a shows the image of the interference-free echo signal. We mixed this echo signal with some simulated interference signals to acquire the contaminated echo signal.

Figure 10b was obtained by applying an imaging algorithm to the contaminated echo signal without interference suppression. It can be noted that the image of the ground target was overshadowed by the interference with bright lines. The Time–frequency filtering method was performed on the contaminated echo signal to achieve interference suppression, and then the imaging algorithm was applied to the signal after interference suppression to obtain Figure 10c. Owing to the Gibbs effect, this algorithm does not suppress the interference well, and it causes a large loss of resolution. Figure 10d presents the image produced by applying the proposed method and imaging algorithm. The steps of this process were as follows: First, based on Equations (4) and (5), the simulated signals were generated to train an SSD network. Second, STFT was used to obtain the TFG of each radar pulse. Then, these TFGs were input to the trained SSD network to detect the existence of interference and to identify the types of interference. Based on this, the interference was suppressed by employing an adaptive filter. Finally, the image was obtained by applying the imaging algorithm to the signal after interference suppression. Compared with Figure 10c, Figure 10d indicates that the interference was removed effectively with a small loss of target information with the proposed method.

## 5. Conclusions

For the purpose of suppressing the multiclass radio frequency interference in SAR echoes and improving the quality of SAR image, a novel method for RFI detection and mitigation based on the SSD deep learning network was proposed in this study. In this method, first, an echo-interference dataset was created and applied to train the SSD network. The time–frequency form of the dataset was acquired by utilizing STFT. After training, a network that has the ability to detect, identify, and estimate the interference was obtained. Next, an echo signal was input to the network in a pulse-by-pulse manner, and the network gave a prediction result, based on which the interference signal can be precisely reconstructed. Finally, the interference signal was mitigated from the target signal by an adaptive filter. The interference suppression results from simulated data proved the validity of this method.

When the SAR echo signal is contaminated by various types of interference signals, even if the positions of these interference signals in the time–frequency domain are known in advance, it is difficult to obtain a good interference suppression effect by conventional methods, such as the time–frequency filtering method. Unlike most of the traditional interference suppression methods, the proposed method uses deep learning technology to extract high-dimensional information of the target and interference signals. Moreover, the proposed method focuses on the time–frequency features of the echo data instead of their corresponding image features, which means that the interference can be detected and identified by relying on SAR echo signals without imaging processing. In future work, we will improve the interference suppression performance of this method by using a large amount of real SAR data, and we will also study suppression methods for other types of interference.

## Figures and Tables

**Table 1:** The network structure of VGG16 convolutional neural network used in this paper.

Layer	Kernel Size	Stride	Padding	Kernel Initialization	Activation
Conv2D	3 × 3	1×1	Same	he_normal	Relu
Conv2D	3×3	1×1	Same	he_normal	Relu
Maxpool	2×2	2×2	Same	None	None
Conv2D	3×3	1×1	Same	he_normal	Relu
Conv2D	3×3	1×1	Same	he_normal	Relu
Maxpool	2×2	2×2	Same	None	None
Conv2D	3×3	1×1	Same	he_normal	Relu
Conv2D	3×3	1×1	Same	he_normal	Relu
Conv2D	3×3	1×1	Same	he_normal	Relu
Maxpool	2×2	2×2	Same	None	None
Conv2D	3×3	1×1	Same	he_normal	Relu
Conv2D	3×3	1×1	Same	he_normal	Relu
Conv2D	3×3	1×1	Same	he_normal	Relu
Maxpool	2×2	2×2	Same	None	None
Conv2D	3×3	1×1	Same	he_normal	Relu
Conv2D	3×3	1×1	Same	he_normal	Relu
Conv2D	3×3	1×1	Same	he_normal	Relu
Maxpool	3×3	1×1	Same	None	None

**Table 2:** Parameters of the target signals.

Parameter	Value
Bandwidth (MHz)	50
Sampling Frequency (MHz)	100
Pulse Width (s)	2.5 × 10${}^{-6}$
Chirp Rate (Hz/s)	20 × 10${}^{12}$
Receive Window Width (s)	10 × 10${}^{-6}$
Signal-to-noise Ratio (dB)	−5
Amplitude Range (dB) ^1^	0–20

${}^{1}$ Indicates the quantized amplitude range.

**Table 3:** Parameters of the interference signals.

Type of Interference	Parameters	Value
RFNI	Range of Frequency (MHz)	0–50 (Uniform Distribution)
	Range of Pulse Width (μs)	1.25–1.66 (Uniform Distribution)
	Amplitude Range (dB) ^1^	30–40 (Rayleigh Distribution)
NBLFMI	Range of Bandwidth (MHz)	0.01–0.5 (Uniform Distribution)
	Range of Pulse Width (μs)	1.25–1.66 (Uniform Distribution)
	Amplitude Range (dB) ^1^	30–40 (Uniform Distribution)
WBLFMI	Range of Bandwidth (MHz)	5–10 (Uniform Distribution)
	Range of Pulse Width (μs)	1.25–1.66 (Uniform Distribution)
	Amplitude Range (dB) ^1^	30–40 (Uniform Distribution)

${}^{1}$ Indicates the quantized amplitude range.

**Table 4:** Estimated parameters of the interference signals.

Type of Interference	Estimated Parameters	Value
RFNI-1	Frequency (MHz) ^1^	15.623
	Pulse Width (s)	1.386 × 10${}^{-6}$
	Time Shift (s)	4.334 × 10${}^{-7}$
RFNI-2	Frequency (MHz) ^1^	33.129
	Pulse Width (s)	1.647 × 10${}^{-6}$
	Time Shift (s)	2.727 × 10${}^{-6}$
NBLFMI	Chirp Rate (Hz/s)	2.806 × 10${}^{12}$
	Pulse Width (s)	1.587 × 10${}^{-6}$
	Time Shift (s)	3.851 × 10${}^{-6}$
WBLFMI	Chirp Rate (Hz/s)	9.445 × 10${}^{12}$
	Pulse Width (s)	1.2955 × 10${}^{-6}$
	Time Shift (s)	7.068 × 10${}^{-7}$

${}^{1}$ Indicates the frequency of the signal after zero-intermediate frequency processing.

**Table 5:** Performance comparison of the two interference suppression methods.

	Interference-Free Signal	Contaminated Signal	Time–Frequency Filtering Method	Proposed Method
**SINR (dB)** ^1^	−5.297	−25.017	−16.399	−15.573
**PSLR (dB)** ^2^	−10.813	None	−7.478	−10.085

${}^{1}$ Reflects the ratio of the energy of target signal to the energy of interference and noise. ${}^{2}$ Reflects the ratio of the peak of the highest secondary lobe to the peak of main lobe.
